# Methyl 2-(3a,8a-dimethyl-4-oxodeca­hydro­azulen-6-yl)acrylate

**DOI:** 10.1107/S1600536811029928

**Published:** 2011-07-30

**Authors:** Mohamed Tebbaa, Ahmed Benharref, Moha Berraho, Jean Claude Daran, Mohamed Akssira, Ahmed Elhakmaoui

**Affiliations:** a Laboratoire de Chimie Biomoleculaire, Substances Naturelles et Réactivité URAC16, Faculté des Sciences Semlalia, BP 2390 Boulevard My Abdellah, 40000 Marrakech, Morocco; b Laboratoire de Chimie de Coordination, 205 Route de Narbonne, 31077 Toulouse Cedex 04, France; c Laboratoire de Chimie Bioorganique et Analytique, URAC 22, BP 146, FSTM, Université Hassan II, Mohammedia–Casablanca 20810 Mohammedia, Morocco

## Abstract

The title compound, C_16_H_24_O_3_, was synthesized from ilicic acid, which was isolated from the aerial part of *Inula viscosa­* (L) Aiton [or *Dittrichia viscosa­* (L) Greuter]. The asymmetric unit contains two independent mol­ecules, in each of which the seven-membered ring shows a chair conformation, whereas the five-membered ring presents disorder. In the two molecules, three C atoms in the five-membered ring are disordered over two positions with site-occupancy factors of 0.53/0.47 and 0.83/0.17. The dihedral angle between the two rings is different in the two mol­ecules [31.7 (3) and 47.7 (7)°]. The crystal structure is stabilized by weak inter­molecular C—H⋯O hydrogen-bond inter­actions.

## Related literature

For background to the medicinal inter­est in *Inula viscosa­* (L) Aiton [or *Dittrichia viscosa­* (L) Greuter], see: Shtacher & Kasshman (1970 ▶); Chiappini *et al.* (1982 ▶); Azoulay *et al.* (1986 ▶); Bohlman *et al.* (1977 ▶); Ceccherelli *et al.* (1988 ▶); Geissman & Toribio (1967 ▶). For conformational analysis, see: Cremer & Pople (1975 ▶). For a related synthesis, see: Barrero *et al.* (2009 ▶).
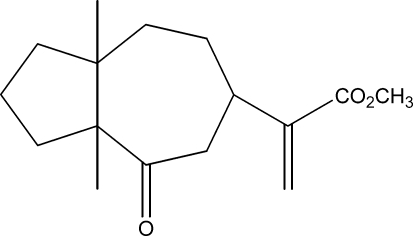


## Experimental

### 

#### Crystal data

C_16_H_24_O_3_
*M* *_r_* = 264.35
Monoclinic, 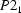

*a* = 6.6954 (3) Å
*b* = 6.9447 (3) Å
*c* = 31.6168 (18) Å
β = 90.095 (7)°
*V* = 1470.10 (12) Å^3^
*Z* = 4
Mo *K*α radiation
μ = 0.08 mm^−1^
*T* = 180 K
0.33 × 0.23 × 0.15 mm

#### Data collection

Agilent Xcalibur Eos Gemini ultra diffractometer
Absorption correction: multi-scan (*CrysAlis PRO*; Agilent, 2010 ▶) *T*_min_ = 0.843, *T*_max_ = 1.000
9032 measured reflections
5575 independent reflections
4990 reflections with *I* > 2σ(*I*)
*R*_int_ = 0.020

#### Refinement

*R*[*F* ^2^ > 2σ(*F* ^2^)] = 0.042
*wR*(*F* ^2^) = 0.110
*S* = 1.06
5575 reflections
356 parameters
19 restraints
H-atom parameters constrained
Δρ_max_ = 0.14 e Å^−3^
Δρ_min_ = −0.19 e Å^−3^

### 

Data collection: *CrysAlis PRO* (Agilent, 2010 ▶); cell refinement: *CrysAlis PRO*; data reduction: *CrysAlis PRO*; program(s) used to solve structure: *SHELXS97* (Sheldrick, 2008 ▶); program(s) used to refine structure: *SHELXL97* (Sheldrick, 2008 ▶); molecular graphics: *ORTEPIII* (Burnett & Johnson, 1996 ▶) and *ORTEP-3 for Windows* (Farrugia, 1997 ▶); software used to prepare material for publication: *WinGX* (Farrugia, 1999 ▶ and *PLATON* (Spek, 2009 ▶).

## Supplementary Material

Crystal structure: contains datablock(s) I, global. DOI: 10.1107/S1600536811029928/om2451sup1.cif

Structure factors: contains datablock(s) I. DOI: 10.1107/S1600536811029928/om2451Isup2.hkl

Supplementary material file. DOI: 10.1107/S1600536811029928/om2451Isup3.cml

Additional supplementary materials:  crystallographic information; 3D view; checkCIF report

## Figures and Tables

**Table 1:** Hydrogen-bond geometry (Å, °)

*D*—H⋯*A*	*D*—H	H⋯*A*	*D*⋯*A*	*D*—H⋯*A*
C112—H11*B*⋯O11^i^	0.93	2.42	3.325 (7)	165
C212—H21*A*⋯O21^i^	0.93	2.45	3.348 (6)	162
C26—H26*B*⋯O23^ii^	0.97	2.58	3.427 (6)	146

Symmetry codes: (i) 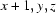; (ii) 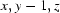.

**Fig. 1.**
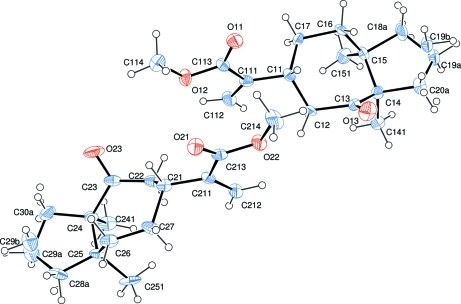
: Molecular structure of the title compound with the atom-labelling scheme. Displacement ellipsoids are drawn at the 30% probability level. H atoms are represented as small spheres of arbitrary radii.

**Fig. 2.**
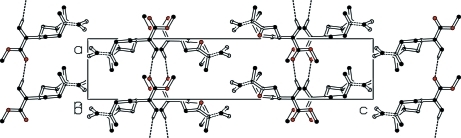
: packing view showing the C–H···O hydrogen bonds as dashed lines. H atoms not involved in hydrogen bonding have been omitted for clarity. [Symmetry codes: (i) x + 1, y, z; (ii) x, y - 1, z.].

**Fig. 3.**
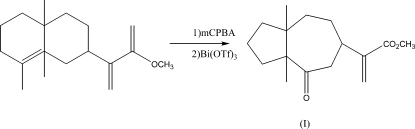
: Synthesis of the title compound.

## supplementary crystallographic information

### Comment

The *Inula Viscosa (L)* is widespread in Mediterranean area and extends to the Atlantic cost of Morocco. It is a well known medicinal plant (Shtacher & Kasshman, 1970; Chiappini *et al.*, 1982) and has some pharmacological activities (Azoulay *et al.*, 1986). This plant has been the subject of chemical investigation in terms of isolating sesquiterpene lactones (Bohlman *et al.*, 1977), sesquiterpene acids (Ceccherelli *et al.*, 1988; Geissman & Toribio, 1967). The ilicic acid is one of the main components of the dichloromethane extract of the *Inula Viscosa (L) Aiton or Dittrichia Viscosa (L) Greuter]*. The literature report one article on the transformation of the ilicic acid (Barrero *et al.*, 2009). In order to prepare products with high added value, that can be used in the pharmacology and cosmetics industry, we have studied the reactivity of this acid. Thus, from the ilicic acid, we have prepared by the method of Barrero *et al.* (2009), 2-(4a,8-Dimethyl-1, 2,3,4,4 a,5,6,7- octahydro-naphthalene -2-yl)-acrylic acid methyl ester. The epoxidation of the latter by metachloroperbenzoic acid (mCPBA), followed by the opening of the epoxide obtained by Bi(OTf)3 leads to 2- (3a,8a-Dimethyl-4-oxo-decahydro-azulene-6- yl)-acrylic acid methyl ester with a yield of 50% (see figure 3). The structure of this new derivative (I) of ilicic acid was confirmed by its single-crystal X-ray structure. The asymmetric unit contains two crystallographically independent molecules (Fig.1). Each molecule is built up from two fused five and seven-membered rings.The seven membered ring shows a chair conformation as indicated by Cremer & Pople (1975) puckering parameters QT = 0.7918 (48) Å, θ2 = 38.41 (34)°, φ2 = -33.23 (58)° and φ3 = 171.04 (52)° for the ring (C21, C22···C27)and QT = 0.8658 (51) Å, θ2 = 39.31 (31)°, φ2 = -32.67 (53)° and φ3 = 173.97 (45)° for the other ring (C11,C12···C17). In the first molecule (C11 to C151), the dihedral angle between the rings is 31.7 (3)°. The corresponding value in the second molecule (C21 to C251) is 47.7 (7)°. In the crystal structure, the molecules are linked by C—H···O intermolecular hydrogen bonds into a chains along the *a* axis (Fig.2).

### Experimental

To 3 g (12 mmol) of 2-(4a,8-Dimethyl-1,2,3,4,4a,5,6,7-octahydro- naphthalen-2-yl)-acrylic acid methyl ester dissolved in 40 ml of dichloromethane was added one equivalent of *m*-chloroperbenzoic acid at 70%. The reaction mixture was stirred at room temperature for 3 h, then treated three times with a solution of sodium bisulfite at 10%. The organic layer was then washed with distilled water three times until neutralization, dried over sodium sulfate, filtered and concentrated under reduced pressure. The residue obtained was chromatographed on silica gel eluting with hexane/ ethyl acetate (98/2) to give quantitatively the corresponding epoxide. 2.5 g (9.4 mmol) of this epoxyde is dissolved with 5% Bi(OTf)3 in 20 ml of dichloromethane. The reaction mixture was left stirring for a period of half an hour and then treated with 10 ml of a solution of sodium bicarbonate to 10%. The organic layer was dried filtered and concentrated under reduced pressure. Chromatography on silica gel, eluting with hexane/ethyl acetate (98/2) of the residue obtained, allowed us to obtain 1.24 g (4.71 mmol) of 2-(3a, 8a-dimethyl-4-oxo-azulene-decahydro-6-yl)-acrylic acid methyl ester. The title compound was recrystallized in dichloromethane.

### Refinement

All H atoms attached to C atoms were fixed geometrically and treated as riding with C—H = 0.96 Å (methyl), 0.97 Å (methylene), 0.98Å (methine) and 0.93 Å (C=CH_2_) with *U*_iso_(H) = 1.2*U*_eq_(C) or *U*_iso_(H) = 1.5*U*_eq_(C_methyl_). In the absence of significant anomalous scattering, the absolute configuration could not be reliably determined, and thus any references to the Flack parameter were removed.

Carbons C18/C19/C20 and C28/C29/C30 of the five membered rings are disordered over two positions. For both molecules, the site occupancy factor of each conformation were refined while restraining their sum to unity. The occupancy factors were found to be equal to 0.53/0.47 for the first molecule, and 0.83/0.17 for the second molecule. Similarity restraints (SAME) were applied to the chemically equivalent bond lengths and angles involving all disordered atoms, while disordered atoms were restrained to have similar atomic displacement parameters within a tolerance s.u. of 0.01 Å^2^ as those of neighbouring atoms.

The structure is a pseudo-merohedral twin with twin law (1 0 0 0 -1 0 0 0 -1) and twin parameter 0.503 (3).

### Crystal data

**Table:**

C_16_H_24_O_3_	*F*(000) = 576
*M**_r_* = 264.35	*D*_x_ = 1.194 Mg m^−^^3^
Monoclinic, *P*2_1_	Mo *K*α radiation, λ = 0.7107 Å
Hall symbol: P 2yb	Cell parameters from 3361 reflections
*a* = 6.6954 (3) Å	θ = 3.6–29.2°
*b* = 6.9447 (3) Å	µ = 0.08 mm^−^^1^
*c* = 31.6168 (18) Å	*T* = 180 K
β = 90.095 (7)°	Block, colourless
*V* = 1470.10 (12) Å^3^	0.33 × 0.23 × 0.15 mm
*Z* = 4	

### Data collection

**Table:**

Agilent Xcalibur Eos Gemini ultra diffractometer	5575 independent reflections
Radiation source: Enhance (Mo) X-ray Source	4990 reflections with *I* > 2σ(*I*)
graphite	*R*_int_ = 0.020
Detector resolution: 16.1978 pixels mm^-1^	θ_max_ = 26.4°, θ_min_ = 3.6°
ω scans	*h* = −8→8
Absorption correction: multi-scan (*CrysAlis PRO*; Agilent, 2010)	*k* = −8→8
*T*_min_ = 0.843, *T*_max_ = 1.000	*l* = −36→39
9032 measured reflections	

### Refinement

**Table:**

Refinement on *F*^2^	Primary atom site location: structure-invariant direct methods
Least-squares matrix: full	Secondary atom site location: difference Fourier map
*R*[*F*^2^ > 2σ(*F*^2^)] = 0.042	Hydrogen site location: inferred from neighbouring sites
*wR*(*F*^2^) = 0.110	H-atom parameters constrained
*S* = 1.06	*w* = 1/[σ^2^(*F*_o_^2^) + (0.058*P*)^2^ + 0.0579*P*] where *P* = (*F*_o_^2^ + 2*F*_c_^2^)/3
5575 reflections	(Δ/σ)_max_ = 0.011
356 parameters	Δρ_max_ = 0.14 e Å^−^^3^
19 restraints	Δρ_min_ = −0.19 e Å^−^^3^

### Special details

**Table:**

Experimental. The crystal is twinned by pseudo-merohedry. The unit cell is monoclinic but it emulates an orthorhombic P 21 21 2 cell.Empirical absorption correction using spherical harmonics, implemented in SCALE3 ABSPACK scaling algorithm. CrysAlisPro (Agilent,2010)
Geometry. All s.u.'s (except the s.u. in the dihedral angle between two l.s. planes) are estimated using the full covariance matrix. The cell s.u.'s are taken into account individually in the estimation of s.u.'s in distances, angles and torsion angles; correlations between s.u.'s in cell parameters are only used when they are defined by crystal symmetry. An approximate (isotropic) treatment of cell s.u.'s is used for estimating s.u.'s involving l.s. planes.

### Fractional atomic coordinates and isotropic or equivalent isotropic displacement parameters (Å^2^)

**Table:**

	*x*	*y*	*z*	*U*_iso_*/*U*_eq_	Occ. (<1)
O11	−0.3396 (6)	0.7937 (7)	0.78385 (10)	0.0491 (9)	
O12	−0.1779 (6)	0.7707 (7)	0.72197 (8)	0.0441 (9)	
O13	0.0462 (8)	0.4408 (6)	0.90038 (11)	0.0603 (11)	
C11	0.0245 (7)	0.7490 (7)	0.83087 (10)	0.0320 (10)	
H11	−0.1000	0.6868	0.8397	0.038*	
C12	0.1966 (8)	0.6169 (6)	0.84329 (11)	0.0370 (11)	
H12A	0.3233	0.6815	0.8388	0.044*	
H12B	0.1943	0.5008	0.8262	0.044*	
C13	0.1702 (9)	0.5650 (7)	0.89132 (14)	0.0376 (11)	
C16	0.0506 (8)	0.9418 (7)	0.90041 (11)	0.0344 (10)	
H16A	0.0105	1.0672	0.9109	0.041*	
H16B	−0.0453	0.8491	0.9111	0.041*	
C17	0.0323 (8)	0.9457 (7)	0.85143 (11)	0.0371 (11)	
H17A	−0.0878	1.0159	0.8439	0.045*	
H17B	0.1453	1.0158	0.8400	0.045*	
C14	0.2954 (9)	0.6686 (7)	0.92227 (13)	0.0375 (11)	
C15	0.2545 (8)	0.8936 (7)	0.91989 (13)	0.0365 (11)	
C18A	0.2343 (11)	0.9472 (10)	0.96834 (13)	0.068 (2)	0.53
H18A	0.3233	1.0532	0.9750	0.082*	0.53
H18B	0.0986	0.9879	0.9743	0.082*	0.53
C19A	0.2859 (15)	0.7758 (13)	0.99513 (19)	0.0438 (13)	0.53
H19A	0.2078	0.7746	1.0210	0.053*	0.53
H19B	0.4267	0.7756	1.0024	0.053*	0.53
C20A	0.2344 (11)	0.6059 (9)	0.96745 (13)	0.0562 (16)	0.53
H20A	0.0926	0.5778	0.9688	0.067*	0.53
H20B	0.3082	0.4924	0.9761	0.067*	0.53
C18B	0.2343 (11)	0.9472 (10)	0.96834 (13)	0.068 (2)	0.47
H18C	0.3638	0.9789	0.9802	0.082*	0.47
H18D	0.1462	1.0569	0.9719	0.082*	0.47
C19B	0.1550 (14)	0.7852 (12)	0.9882 (2)	0.0438 (13)	0.47
H19C	0.0105	0.7874	0.9861	0.053*	0.47
H19D	0.1911	0.7858	1.0179	0.053*	0.47
C20B	0.2344 (11)	0.6059 (9)	0.96745 (13)	0.0562 (16)	0.47
H20C	0.1324	0.5068	0.9664	0.067*	0.47
H20D	0.3488	0.5565	0.9828	0.067*	0.47
C111	0.0153 (7)	0.7762 (8)	0.78377 (12)	0.0350 (10)	
C112	0.1773 (8)	0.7999 (10)	0.75949 (14)	0.0527 (14)	
H11A	0.1632	0.8188	0.7305	0.063*	
H11B	0.3039	0.7974	0.7716	0.063*	
C113	−0.1814 (7)	0.7805 (8)	0.76432 (12)	0.0340 (10)	
C114	−0.3611 (8)	0.7761 (8)	0.69985 (12)	0.0453 (11)	
H11C	−0.4307	0.8930	0.7066	0.068*	
H11D	−0.4413	0.6676	0.7079	0.068*	
H11E	−0.3359	0.7715	0.6700	0.068*	
C141	0.5147 (9)	0.6146 (9)	0.91621 (15)	0.0562 (16)	
H14A	0.5558	0.6476	0.8880	0.084*	
H14B	0.5311	0.4786	0.9205	0.084*	
H14C	0.5951	0.6835	0.9363	0.084*	
C151	0.4187 (10)	1.0125 (9)	0.90107 (18)	0.0537 (15)	
H15A	0.3809	1.1459	0.9015	0.081*	
H15B	0.4417	0.9726	0.8724	0.081*	
H15C	0.5386	0.9954	0.9173	0.081*	
O21	−0.3368 (5)	0.2891 (7)	0.71830 (9)	0.0455 (8)	
O22	−0.1755 (6)	0.3099 (7)	0.78059 (8)	0.0435 (8)	
O23	0.0557 (8)	0.6369 (6)	0.60233 (12)	0.0710 (13)	
C21	0.0213 (7)	0.3281 (7)	0.67099 (11)	0.0336 (10)	
H21	−0.1050	0.3890	0.6628	0.040*	
C22	0.1921 (8)	0.4596 (6)	0.65588 (14)	0.0435 (12)	
H22A	0.1943	0.5748	0.6732	0.052*	
H22B	0.3183	0.3933	0.6598	0.052*	
C23	0.1736 (7)	0.5141 (6)	0.61251 (15)	0.0367 (10)	
C26	0.0504 (8)	0.1303 (7)	0.60083 (14)	0.0422 (11)	
H26A	−0.0491	0.2194	0.5902	0.051*	
H26B	0.0119	0.0029	0.5913	0.051*	
C27	0.0351 (8)	0.1312 (7)	0.64743 (13)	0.0423 (12)	
H27A	0.1503	0.0631	0.6585	0.051*	
H27B	−0.0820	0.0565	0.6551	0.051*	
C24	0.2925 (8)	0.4082 (7)	0.57727 (13)	0.0343 (10)	
C25	0.2532 (7)	0.1810 (6)	0.57876 (14)	0.0364 (11)	
C28A	0.2445 (10)	0.1255 (7)	0.53289 (15)	0.0547 (15)	0.83
H28A	0.3450	0.0283	0.5271	0.066*	0.83
H28B	0.1146	0.0707	0.5266	0.066*	0.83
C29A	0.2795 (15)	0.2957 (12)	0.50553 (16)	0.0775 (19)	0.83
H29A	0.4178	0.3013	0.4965	0.093*	0.83
H29B	0.1943	0.2918	0.4807	0.093*	0.83
C30A	0.2296 (11)	0.4625 (8)	0.53251 (15)	0.0566 (17)	0.83
H30A	0.0874	0.4888	0.5314	0.068*	0.83
H30B	0.3010	0.5763	0.5231	0.068*	0.83
C28B	0.2445 (10)	0.1255 (7)	0.53289 (15)	0.0547 (15)	0.17
H28C	0.3776	0.1080	0.5214	0.066*	0.17
H28D	0.1693	0.0073	0.5291	0.066*	0.17
C29B	0.143 (5)	0.289 (3)	0.5125 (7)	0.0775 (19)	0.17
H29C	0.1674	0.2891	0.4822	0.093*	0.17
H29D	−0.0002	0.2830	0.5173	0.093*	0.17
C30B	0.2296 (11)	0.4625 (8)	0.53251 (15)	0.0566 (17)	0.17
H30C	0.1315	0.5653	0.5333	0.068*	0.17
H30D	0.3443	0.5067	0.5165	0.068*	0.17
C211	0.0190 (7)	0.3028 (7)	0.71832 (11)	0.0325 (10)	
C212	0.1757 (7)	0.2744 (9)	0.74169 (12)	0.0445 (12)	
H21A	0.3016	0.2685	0.7293	0.053*	
H21B	0.1621	0.2601	0.7708	0.053*	
C213	−0.1856 (7)	0.2982 (7)	0.73821 (13)	0.0333 (9)	
C214	−0.3691 (8)	0.2993 (11)	0.80196 (13)	0.0582 (15)	
H21C	−0.4298	0.1767	0.7963	0.087*	
H21D	−0.4542	0.4003	0.7917	0.087*	
H21E	−0.3503	0.3138	0.8319	0.087*	
C241	0.5158 (7)	0.4632 (8)	0.58597 (18)	0.0523 (14)	
H24A	0.5556	0.4133	0.6130	0.078*	
H24B	0.5295	0.6008	0.5860	0.078*	
H24C	0.5990	0.4091	0.5643	0.078*	
C251	0.4216 (10)	0.0690 (8)	0.6025 (2)	0.0612 (16)	
H25A	0.5398	0.0658	0.5854	0.092*	
H25B	0.3779	−0.0603	0.6081	0.092*	
H25C	0.4506	0.1325	0.6288	0.092*	

### Atomic displacement parameters (Å^2^)

**Table:**

	*U*^11^	*U*^22^	*U*^33^	*U*^12^	*U*^13^	*U*^23^
O11	0.0296 (16)	0.068 (2)	0.0497 (16)	0.0131 (19)	−0.0007 (16)	−0.0057 (19)
O12	0.0329 (16)	0.067 (2)	0.0324 (11)	−0.0016 (19)	−0.0084 (14)	0.0003 (16)
O13	0.069 (3)	0.045 (2)	0.067 (2)	−0.020 (2)	−0.003 (2)	0.0090 (18)
C11	0.026 (2)	0.040 (3)	0.0300 (17)	−0.0028 (19)	0.0027 (16)	−0.0067 (17)
C12	0.046 (3)	0.039 (3)	0.0263 (16)	0.014 (3)	−0.0010 (19)	−0.0137 (17)
C13	0.054 (3)	0.0261 (19)	0.0330 (18)	0.006 (2)	0.001 (2)	0.0034 (15)
C16	0.037 (2)	0.038 (3)	0.0281 (18)	0.006 (2)	0.0088 (19)	−0.0104 (17)
C17	0.043 (3)	0.037 (2)	0.0315 (18)	0.009 (2)	0.0012 (19)	0.0118 (18)
C14	0.039 (3)	0.038 (3)	0.0361 (19)	−0.001 (3)	0.003 (2)	0.0045 (18)
C15	0.043 (3)	0.043 (2)	0.0235 (16)	−0.004 (2)	0.0041 (18)	−0.0050 (16)
C18A	0.077 (5)	0.103 (4)	0.0249 (19)	0.025 (4)	−0.011 (2)	−0.004 (2)
C19A	0.049 (3)	0.056 (3)	0.026 (2)	0.000 (4)	−0.003 (2)	0.003 (3)
C20A	0.069 (4)	0.066 (4)	0.034 (2)	0.002 (3)	0.001 (3)	0.000 (2)
C18B	0.077 (5)	0.103 (4)	0.0249 (19)	0.025 (4)	−0.011 (2)	−0.004 (2)
C19B	0.049 (3)	0.056 (3)	0.026 (2)	0.000 (4)	−0.003 (2)	0.003 (3)
C20B	0.069 (4)	0.066 (4)	0.034 (2)	0.002 (3)	0.001 (3)	0.000 (2)
C111	0.029 (2)	0.034 (2)	0.042 (2)	0.0037 (19)	−0.0016 (18)	−0.007 (2)
C112	0.034 (3)	0.083 (4)	0.0409 (18)	0.019 (3)	0.008 (2)	−0.006 (2)
C113	0.034 (2)	0.035 (2)	0.0333 (17)	−0.002 (2)	0.0013 (19)	−0.0075 (19)
C114	0.043 (3)	0.040 (2)	0.053 (2)	−0.012 (2)	−0.014 (2)	0.003 (2)
C141	0.061 (4)	0.060 (4)	0.047 (2)	0.026 (3)	−0.003 (3)	0.002 (3)
C151	0.052 (3)	0.052 (3)	0.057 (3)	−0.004 (3)	−0.004 (3)	0.008 (2)
O21	0.0226 (14)	0.067 (2)	0.0464 (15)	0.0066 (18)	0.0044 (14)	0.0054 (18)
O22	0.0311 (15)	0.061 (2)	0.0383 (12)	0.0060 (18)	0.0117 (14)	0.0011 (16)
O23	0.096 (3)	0.0341 (18)	0.083 (2)	0.030 (2)	0.035 (3)	0.0178 (18)
C21	0.0238 (19)	0.031 (2)	0.046 (2)	−0.0050 (18)	0.0084 (18)	−0.0005 (17)
C22	0.038 (3)	0.027 (2)	0.065 (3)	−0.003 (2)	0.015 (2)	−0.006 (2)
C23	0.030 (2)	0.0210 (18)	0.059 (2)	−0.0053 (18)	0.014 (2)	−0.0034 (16)
C26	0.039 (3)	0.024 (2)	0.064 (3)	−0.009 (2)	0.008 (2)	−0.0007 (19)
C27	0.038 (3)	0.031 (2)	0.057 (2)	−0.013 (2)	0.014 (2)	−0.016 (2)
C24	0.036 (2)	0.028 (2)	0.038 (2)	0.002 (2)	0.012 (2)	0.0003 (16)
C25	0.039 (3)	0.0191 (18)	0.051 (2)	0.0026 (19)	0.012 (2)	−0.0040 (16)
C28A	0.067 (4)	0.033 (2)	0.064 (3)	0.000 (2)	0.005 (3)	−0.025 (2)
C29A	0.106 (5)	0.084 (5)	0.043 (3)	0.007 (5)	0.002 (3)	−0.004 (3)
C30A	0.073 (4)	0.044 (3)	0.052 (2)	0.017 (3)	0.006 (3)	0.018 (2)
C28B	0.067 (4)	0.033 (2)	0.064 (3)	0.000 (2)	0.005 (3)	−0.025 (2)
C29B	0.106 (5)	0.084 (5)	0.043 (3)	0.007 (5)	0.002 (3)	−0.004 (3)
C30B	0.073 (4)	0.044 (3)	0.052 (2)	0.017 (3)	0.006 (3)	0.018 (2)
C211	0.026 (2)	0.037 (2)	0.0346 (19)	0.002 (2)	0.0069 (18)	−0.007 (2)
C212	0.026 (2)	0.069 (3)	0.0380 (17)	0.017 (2)	0.0119 (19)	0.000 (2)
C213	0.024 (2)	0.029 (2)	0.047 (2)	0.0027 (19)	0.0126 (19)	0.0012 (19)
C214	0.036 (3)	0.091 (4)	0.048 (2)	0.000 (3)	0.027 (2)	−0.002 (3)
C241	0.034 (3)	0.045 (3)	0.078 (3)	−0.005 (2)	0.018 (3)	0.000 (3)
C251	0.048 (3)	0.032 (3)	0.104 (4)	0.025 (2)	0.006 (3)	−0.009 (2)

### Geometric parameters (Å, °)

**Table:**

O11—C113	1.229 (6)	O21—C213	1.193 (6)
O12—C113	1.341 (4)	O22—C213	1.344 (4)
O12—C114	1.413 (6)	O22—C214	1.464 (6)
O13—C13	1.231 (7)	O23—C23	1.206 (7)
C11—C111	1.502 (5)	C21—C211	1.507 (5)
C11—C17	1.513 (6)	C21—C22	1.540 (6)
C11—C12	1.524 (6)	C21—C27	1.560 (6)
C11—H11	0.9800	C21—H21	0.9800
C12—C13	1.571 (5)	C22—C23	1.428 (6)
C12—H12A	0.9700	C22—H22A	0.9700
C12—H12B	0.9700	C22—H22B	0.9700
C13—C14	1.475 (7)	C23—C24	1.554 (6)
C16—C15	1.535 (7)	C26—C27	1.477 (6)
C16—C17	1.554 (5)	C26—C25	1.567 (7)
C16—H16A	0.9700	C26—H26A	0.9700
C16—H16B	0.9700	C26—H26B	0.9700
C17—H17A	0.9700	C27—H27A	0.9700
C17—H17B	0.9700	C27—H27B	0.9700
C14—C141	1.528 (8)	C24—C30A	1.524 (7)
C14—C20A	1.548 (6)	C24—C241	1.567 (8)
C14—C15	1.588 (6)	C24—C25	1.600 (6)
C15—C151	1.498 (8)	C25—C28A	1.502 (6)
C15—C18A	1.582 (5)	C25—C251	1.563 (8)
C18A—C19A	1.501 (10)	C28A—C29A	1.484 (9)
C18A—H18A	0.9700	C28A—H28A	0.9700
C18A—H18B	0.9700	C28A—H28B	0.9700
C19A—C20A	1.509 (10)	C29A—C30A	1.477 (9)
C19A—H19A	0.9700	C29A—H29A	0.9700
C19A—H19B	0.9700	C29A—H29B	0.9700
C20A—H20A	0.9700	C30A—H30A	0.9700
C20A—H20B	0.9700	C30A—H30B	0.9700
C19B—H19C	0.9700	C29B—H29C	0.9700
C19B—H19D	0.9700	C29B—H29D	0.9700
C111—C112	1.339 (7)	C211—C212	1.299 (7)
C111—C113	1.454 (7)	C211—C213	1.508 (6)
C112—H11A	0.9300	C212—H21A	0.9300
C112—H11B	0.9300	C212—H21B	0.9300
C114—H11C	0.9600	C214—H21C	0.9600
C114—H11D	0.9600	C214—H21D	0.9600
C114—H11E	0.9600	C214—H21E	0.9600
C141—H14A	0.9600	C241—H24A	0.9600
C141—H14B	0.9600	C241—H24B	0.9600
C141—H14C	0.9600	C241—H24C	0.9600
C151—H15A	0.9600	C251—H25A	0.9600
C151—H15B	0.9600	C251—H25B	0.9600
C151—H15C	0.9600	C251—H25C	0.9600
			
C113—O12—C114	118.6 (4)	C213—O22—C214	114.3 (4)
C111—C11—C17	108.3 (4)	C211—C21—C22	112.6 (4)
C111—C11—C12	111.2 (3)	C211—C21—C27	111.9 (4)
C17—C11—C12	114.0 (4)	C22—C21—C27	109.1 (3)
C111—C11—H11	107.7	C211—C21—H21	107.7
C17—C11—H11	107.7	C22—C21—H21	107.7
C12—C11—H11	107.7	C27—C21—H21	107.7
C11—C12—C13	107.6 (4)	C23—C22—C21	113.0 (4)
C11—C12—H12A	110.2	C23—C22—H22A	109.0
C13—C12—H12A	110.2	C21—C22—H22A	109.0
C11—C12—H12B	110.2	C23—C22—H22B	109.0
C13—C12—H12B	110.2	C21—C22—H22B	109.0
H12A—C12—H12B	108.5	H22A—C22—H22B	107.8
O13—C13—C14	124.8 (4)	O23—C23—C22	120.0 (4)
O13—C13—C12	117.5 (5)	O23—C23—C24	118.6 (4)
C14—C13—C12	117.7 (4)	C22—C23—C24	121.2 (4)
C15—C16—C17	118.3 (4)	C27—C26—C25	120.2 (4)
C15—C16—H16A	107.7	C27—C26—H26A	107.3
C17—C16—H16A	107.7	C25—C26—H26A	107.3
C15—C16—H16B	107.7	C27—C26—H26B	107.3
C17—C16—H16B	107.7	C25—C26—H26B	107.3
H16A—C16—H16B	107.1	H26A—C26—H26B	106.9
C11—C17—C16	114.6 (4)	C26—C27—C21	119.0 (4)
C11—C17—H17A	108.6	C26—C27—H27A	107.6
C16—C17—H17A	108.6	C21—C27—H27A	107.6
C11—C17—H17B	108.6	C26—C27—H27B	107.6
C16—C17—H17B	108.6	C21—C27—H27B	107.6
H17A—C17—H17B	107.6	H27A—C27—H27B	107.0
C13—C14—C141	110.1 (4)	C30A—C24—C23	114.0 (4)
C13—C14—C20A	109.0 (4)	C30A—C24—C241	111.5 (4)
C141—C14—C20A	107.5 (5)	C23—C24—C241	104.3 (4)
C13—C14—C15	110.5 (4)	C30A—C24—C25	103.1 (4)
C141—C14—C15	113.6 (5)	C23—C24—C25	111.2 (4)
C20A—C14—C15	106.0 (4)	C241—C24—C25	113.0 (4)
C151—C15—C16	111.9 (4)	C28A—C25—C251	111.4 (4)
C151—C15—C18A	108.5 (5)	C28A—C25—C26	109.8 (4)
C16—C15—C18A	105.1 (4)	C251—C25—C26	107.4 (4)
C151—C15—C14	115.8 (5)	C28A—C25—C24	103.4 (4)
C16—C15—C14	112.8 (4)	C251—C25—C24	112.8 (5)
C18A—C15—C14	101.6 (4)	C26—C25—C24	112.2 (4)
C19A—C18A—C15	109.9 (5)	C29A—C28A—C25	110.6 (4)
C19A—C18A—H18A	109.7	C29A—C28A—H28A	109.5
C15—C18A—H18A	109.7	C25—C28A—H28A	109.5
C19A—C18A—H18B	109.7	C29A—C28A—H28B	109.5
C15—C18A—H18B	109.7	C25—C28A—H28B	109.5
H18A—C18A—H18B	108.2	H28A—C28A—H28B	108.1
C18A—C19A—C20A	103.9 (5)	C30A—C29A—C28A	104.6 (4)
C18A—C19A—H19A	111.0	C30A—C29A—H29A	110.8
C20A—C19A—H19A	111.0	C28A—C29A—H29A	110.8
C18A—C19A—H19B	111.0	C30A—C29A—H29B	110.8
C20A—C19A—H19B	111.0	C28A—C29A—H29B	110.8
H19A—C19A—H19B	109.0	H29A—C29A—H29B	108.9
C19A—C20A—C14	104.8 (5)	C29A—C30A—C24	106.2 (5)
C19A—C20A—H20A	110.8	C29A—C30A—H30A	110.5
C14—C20A—H20A	110.8	C24—C30A—H30A	110.5
C19A—C20A—H20B	110.8	C29A—C30A—H30B	110.5
C14—C20A—H20B	110.8	C24—C30A—H30B	110.5
H20A—C20A—H20B	108.9	H30A—C30A—H30B	108.7
H19C—C19B—H19D	108.2	H29C—C29B—H29D	108.8
C112—C111—C113	119.3 (4)	C212—C211—C21	125.1 (4)
C112—C111—C11	123.4 (4)	C212—C211—C213	119.6 (3)
C113—C111—C11	117.3 (4)	C21—C211—C213	115.2 (4)
C111—C112—H11A	120.0	C211—C212—H21A	120.0
C111—C112—H11B	120.0	C211—C212—H21B	120.0
H11A—C112—H11B	120.0	H21A—C212—H21B	120.0
O11—C113—O12	121.3 (4)	O21—C213—O22	124.9 (4)
O11—C113—C111	124.7 (3)	O21—C213—C211	123.5 (3)
O12—C113—C111	113.9 (4)	O22—C213—C211	111.6 (4)
O12—C114—H11C	109.5	O22—C214—H21C	109.5
O12—C114—H11D	109.5	O22—C214—H21D	109.5
H11C—C114—H11D	109.5	H21C—C214—H21D	109.5
O12—C114—H11E	109.5	O22—C214—H21E	109.5
H11C—C114—H11E	109.5	H21C—C214—H21E	109.5
H11D—C114—H11E	109.5	H21D—C214—H21E	109.5
C14—C141—H14A	109.5	C24—C241—H24A	109.5
C14—C141—H14B	109.5	C24—C241—H24B	109.5
H14A—C141—H14B	109.5	H24A—C241—H24B	109.5
C14—C141—H14C	109.5	C24—C241—H24C	109.5
H14A—C141—H14C	109.5	H24A—C241—H24C	109.5
H14B—C141—H14C	109.5	H24B—C241—H24C	109.5
C15—C151—H15A	109.5	C25—C251—H25A	109.5
C15—C151—H15B	109.5	C25—C251—H25B	109.5
H15A—C151—H15B	109.5	H25A—C251—H25B	109.5
C15—C151—H15C	109.5	C25—C251—H25C	109.5
H15A—C151—H15C	109.5	H25A—C251—H25C	109.5
H15B—C151—H15C	109.5	H25B—C251—H25C	109.5

### Hydrogen-bond geometry (Å, °)

**Table:**

*D*—H···*A*	*D*—H	H···*A*	*D*···*A*	*D*—H···*A*
C112—H11B···O11^i^	0.93	2.42	3.325 (7)	165
C212—H21A···O21^i^	0.93	2.45	3.348 (6)	162
C26—H26B···O23^ii^	0.97	2.58	3.427 (6)	146

Symmetry codes: (i) *x*+1, *y*, *z*; (ii) *x*, *y*−1, *z*.
